# Electroacupuncture in Patients With Early Urinary Incontinence After Radical Prostatectomy: A Randomized Clinical Trial

**DOI:** 10.1001/jamanetworkopen.2025.34491

**Published:** 2025-09-30

**Authors:** Jiahui Niu, Yang Wang, Yujuan Wang, Jingyan Shi, Jing Liang, Xiaozhi Zhao, Tianshu Xu, Hongqian Guo, Xuefeng Qiu

**Affiliations:** 1 Department of Urology, Nanjing Drum Tower Hospital, Affiliated Hospital of Medical School, Nanjing University, Nanjing, China; 2 Institute of Urology, Nanjing University, Nanjing, China; 3 Department of Traditional Chinese Medicine, Nanjing Drum Tower Hospital, Nanjing University Medical School, Nanjing, China

## Abstract

**Importance:**

Postprostatectomy urinary incontinence (UI) impairs quality of life, yet effective noninvasive therapies remain limited.

**Objective:**

To investigate the efficacy and safety of electroacupuncture in patients with early UI after radical prostatectomy (RP).

**Design, Setting, and Participants:**

This prospective, single-center, randomized, single-blinded clinical trial was conducted at Nanjing Drum Tower Hospital, Affiliated Hospital of Medical School, Nanjing University, from July 23, 2021, to November 1, 2024, with 20-week follow-up completed March 24, 2025. Men with localized prostate cancer who underwent robot-assisted RP and experienced UI (defined as use of ≥2 pads per day) 4 to 6 weeks postoperatively were consecutively enrolled and randomly assigned 1:1 to the electroacupuncture or sham stimulation group. Patients with any prior neoadjuvant hormonal therapy, any prior prostatic surgery, any history of pelvic surgery, and history of UI before RP were excluded. Three participants withdrew before the intervention, and 101 (91.8%) completed follow-up. Intention-to-treat analysis was used.

**Interventions:**

Patients in the electroacupuncture group received electroacupuncture administered at bilateral Ciliao, Zhongliao, and Xialiao acupoints, with sparse-dense wave electrostimulation (alternating between 2 and 15 Hz) for 30 minutes per session, 3 times per week for 6 weeks. The sham stimulation group underwent identical procedures with nonpuncturing flat-tip needles at offset points.

**Main Outcomes and Measures:**

The primary outcome was urinary continence (UC), defined as the use of 0 to 1 pad per day 6 weeks after the first electroacupuncture session.

**Results:**

A total of 110 men (median age, 69 [IQR, 67-72] years) were randomized. At week 6, the intention-to-treat analysis showed that the electroacupuncture group achieved a significantly higher UC rate than the sham stimulation group (24 of 55 [43.6%] vs 12 of 55 [21.8%]; relative risk, 2.00; 95% CI, 1.12-3.59; *P* = .02).

**Conclusions and Relevance:**

The findings of this randomized clinical trial show that electroacupuncture significantly accelerated postprostatectomy UC recovery and may serve as a safe adjunct to standard care. These findings support integrating electroacupuncture into multimodal rehabilitation protocols to reduce early UI burden.

**Trial Registration:**

ClinicalTrials.gov Identifier: NCT04972669

## Key Points

**Question:**

Does electroacupuncture improve early urinary continence (UC) recovery after radical prostatectomy compared with sham stimulation?

**Findings:**

In this randomized clinical trial of 110 men, electroacupuncture significantly increased the 6-week UC rate (44% vs 22%) and reduced 24-hour urine leakage more effectively than sham treatment, with minimal adverse events.

**Meaning:**

Results of this study suggest that electroacupuncture is a safe and effective noninvasive therapy to accelerate UC recovery after prostatectomy, supporting its integration into postoperative rehabilitation protocols.

## Teaser

This randomized clinical trial investigates the efficacy and safety of electroacupuncture compared with sham stimulation in the treatment of early urinary incontinence among men undergoing radical prostatectomy for prostate cancer.

## Introduction

Radical prostatectomy (RP) is one of the primary treatments for localized prostate cancer, offering more than 85% cancer-specific survival at a 10-year follow-up.^1^ However, postoperative urinary incontinence (UI) is the most common issue faced by patients after RP, with early postoperative UI (3 months postoperatively) occurring in 25% to 86% of patients.^2,3^ This significantly impacts daily quality of life, impeding patients’ societal reintegration and inflicting psychological distress.^4,5^

UI after RP primarily arises from intraoperative damage of the urinary sphincter or its innervation, leading to stress UI (SUI).^6,7^ Current interventions for post-RP UI are limited. Surgical interventions, such as artificial urinary sphincter implantation and male slings carry risks of infection and mechanical dysfunction.^8,9,10,11^ Conservative strategies such as pelvic floor muscle training (PFMT) offer modest benefits,^12^ whereas pharmacological treatments lack robust evidence for their efficacy.^5^ Therefore, the development of novel, noninvasive therapy to address this unmet clinical need remains imperative.

Emerging evidence suggests that sacral nerve stimulation can strengthen the urinary sphincter or pelvic floor muscles, thereby ameliorating SUI.^13,14^ The European Association of Urology guidelines on male UI and nonsurgical management of UI have also highlighted that PFMT combined with pelvic floor muscle electrical stimulation significantly increases the short-term recovery of urinary continence (UC) postoperatively.^15,16^ Electroacupuncture, a potential noninvasive alternative, may exert a similar therapeutic effect through sacral nerve stimulation.^17^ Electroacupuncture not only integrates the concept of pelvic floor muscle electrical stimulation,^17^ it also achieves a higher degree of treatment precision when guided by the principles of traditional Chinese medicine (TCM) acupuncture point theory.^18,19^. Previous studies have demonstrated the efficacy of electroacupuncture for SUI in women.^19,20^ A few studies^21,22^ reported the use of acupuncture for post-RP UI. However, those studies had significant limitations, such as small sample sizes and the absence of a sham control, potentially inflating placebo effects and reducing validity. Due to these limitations, the efficacy and safety of electroacupuncture for men with postprostatectomy UI remains unclear. Therefore, this randomized clinical trial was designed to assess the effectiveness of electroacupuncture for the treatment of UI after RP.

## Methods

### Study Design

This prospective, single-center, single-blinded randomized clinical trial was undertaken in the Nanjing Drum Tower Hospital, Affiliated Hospital of Medical School, Nanjing University, Nanjing, China, and was designed to investigate the efficacy and safety of electroacupuncture at the bilateral Baliao compared with sham stimulation in the treatment of early UI after RP. The trial protocol (found in Supplement 1) was approved by the Review Board of Nanjing Drum Tower Hospital. Written informed consent was obtained from all participants. The report followed the Consolidated Standards of Reporting Trials (CONSORT) reporting guideline.

### Study Population

From July 23, 2021, to November 1, 2024, patients with clinically localized prostate cancer who underwent standard robot-assisted RP at our center and experienced early UI were enrolled in a prospectively registered trial. The surgical procedures were performed with a 4-arm da Vinci System, involving standard robot-assisted RP procedures, which typically include bladder neck dissection, prostatectomy, and anastomosis of the urethra to the bladder neck.^23^ In accordance with the European Association of Urology guidelines,^1^ nerve-sparing techniques were used when oncologically and anatomically feasible to preserve erectile function and potentially enhance UC recovery. Lymph node dissection was also performed. The inclusion criteria consisted of (1) localized prostate cancer (stage cT1-cT2 N0 M0); (2) 4 to 6 weeks after robot-assisted RP; (3) use of a mean of at least 2 pads per 24 hours; (4) prostate-specific antigen level below that denoting cure level 1 month after surgery; and (5) Eastern Cooperative Oncology Group performance status of 0 to 1. Patients with the following characteristics were excluded: (1) any prior neoadjuvant hormonal therapy; (2) any prior prostatic surgery; (3) any history of pelvic surgery; and (4) history of UI before RP. The participants were blinded to the treatment assignment.

### Randomization and Blinding

Randomization was performed by an independent statistician using a computer-generated sequence. The allocation sequence was concealed in sequentially numbered, opaque, sealed envelopes, which were opened by a research assistant only after baseline assessments to ensure allocation concealment. Participants were randomly assigned (1:1 ratio) to receive electroacupuncture or sham stimulation. Participants, outcome assessors, and statistical analysts were blinded to the treatment assignment, but the acupuncturists (Yang Wang and Yujuan Wang) were aware of intervention allocation. Time targets were randomization on the day of enrollment and randomization to intervention initiation within 5 days.

### Interventions

**Figure 1.**
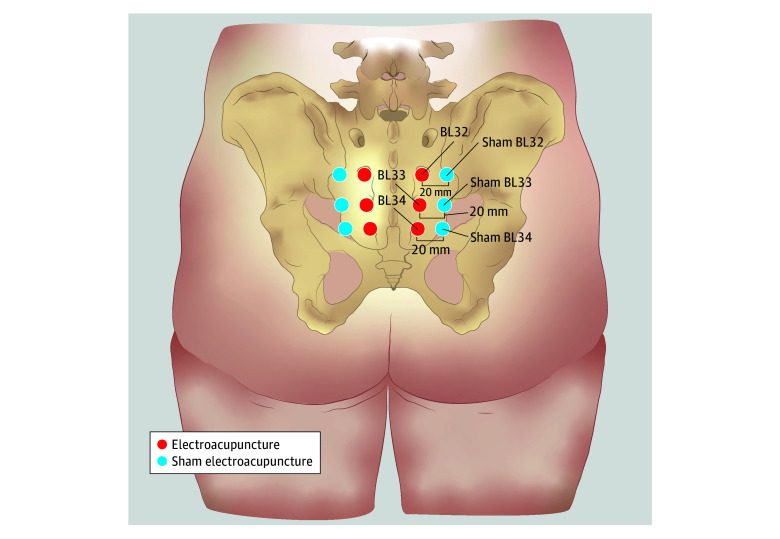
Acupoints Location for the Electroacupuncture Group and Sham Stimulation Group This figure was created by Jiahui Niu, MD, using Adobe Illustrator 2024, version 28.0.

Based on the principles of TCM acupuncture theory and neuroanatomical structures, the acupuncture sites were selected as bilateral Ciliao (BL32), Zhongliao (BL33), and Xialiao (BL34) acupoints on the bladder meridian,^24^ which are closely associated with the sacral micturition center and are located in the second (BL32), third (BL33), and fourth (BL34) sacral foramina (Figure 1).^25,26^ Participants in the experimental group received electroacupuncture stimulation at a total of 6 acupoints, bilaterally at the aforementioned locations. After skin disinfection, sterile adhesive pads were placed on predetermined acupoints. Single-use sterile acupuncture needles with a diameter of 0.30 mm and a length of 75 mm were selected for the procedure. The needles were inserted through the adhesive pads in an inferomedial direction at specific angles. The angle between the needle shaft and the skin surface ranged from 45° to 65°. The optimal angle between the needle shaft and the posterior midline of the body for needle insertion was 20° to 40° for BL34 and 10° to 25° for BL32 and BL33. The optimal depth for electrostimulation varied from cranial to caudal, with depths of 35 to 55 mm for BL32, 25 to 45 mm for BL33, and 20 to 40 mm for BL34. Following needle insertion, small, equal manipulations of twirling, lifting, and thrusting were performed on all needles to reach de qi, a complex sensation characterized by soreness, numbness, swelling, and heaviness and marked by increased resistance to the needle by the acupuncturist. After de qi, the bilateral BL32 (anode) and BL33 (cathode) were connected to the electronic acupuncture therapy device (SDZ-V; Suzhou Medical Supplies Factory Co Ltd). The sparse-dense wave, with a frequency alternating between 2 and 15 Hz, was applied, and the current intensity was adjusted to the maximum tolerable level for the patient, with the needles retained for 30 minutes. Participants received 3 treatment sessions per week (ideally every other day) for 6 consecutive weeks, totaling 18 sessions.

Participants in the control group underwent identical preparation procedures, but with single-use custom-made flat-tip acupuncture needles (diameter, 0.30 mm; length, 75 mm) to penetrate the adhesive pads without skin puncture. The sham acupuncture points were offset approximately 20 mm laterally from the experimental group’s points. No de qi manipulation was performed, and the duration and frequency of current output were the same as in the experimental group. The treatment course and intervals for the control group were consistent with those of the experimental group.

All participants were suggested to undergo PFMT as part of standardized postoperative care. In accordance with our institutional protocol, PFMT was initiated after catheter removal, as recommended by American Urology Association and Society for Urodynamics, Female Pelvic Medicine & Urogenital Reconstruction guideline.^4^ Based on previously reported protocols, participants performed 3 sets of pelvic floor muscle exercises daily, with each set consisting of 10 contractions held for 10 seconds each and a 10-second rest between contractions.^27,28^ The intervention report adhered to the Standards for Reporting Interventions in Clinical Trials of Acupuncture (STRICTA) guidelines.^29^

### Outcome Assessment

Investigators supplied participants with a sufficient quantity of identical urinary pads, weighing devices, and case report forms and instructed the participants on standardized and timely monitoring and documentation of 24-hour urinary pad use and UI status. The patients’ UC recovery was followed up at the end of the treatment course and at 12, 16, and 20 weeks after randomization, completed on March 24, 2025. The primary outcome was UC, defined as the use of 0 to 1 pad per day at 6 weeks after the first electroacupuncture session.^30^ Secondary outcomes included changes in the amount of 24-hour urine leakage, changes in the mean number of pads used daily, prostate-related symptoms assessed by changes in the Expanded Prostate Cancer Index Composite for Clinical Practice (EPIC-CP) UI score at the end of 6-week treatment period compared with baseline, and the time to UC recovery in weeks.

Adverse events (AE) related to acupuncture were documented by both acupuncturists’ records and patient reports and were graded according to the National Cancer Institute Common Terminology Criteria for Adverse Events, version 5.0. The anticipated adverse events primarily encompassed complications associated with acupuncture, including subcutaneous hemorrhage, infection, and abscess caused by needle puncture and the pain induced by needle insertion, and complications arising from electrical stimulation, such as local muscle spasms, local discomfort, and the induction of central nervous system disorders (eg, epilepsy).

### Statistical Analysis

Based on the results of our center’s preliminary records, patients with early UI 1 month postoperatively achieved a UC rate of 48% after 6 weeks of combined treatment with electroacupuncture and PFMT. In contrast, for those who performed PFMT alone, the UC rate was only 20% at 3 months postoperatively. The present study was designed to recruit participants during a 1-year period, with a 6-month follow-up for assessment. We adopted a 2-tailed significance level of *P* < .05 and 80% power, assuming a dropout rate of 10%, with a 1:1 allocation ratio. The sample size was calculated using the Fisher exact test formula with PASS 2021, version 21.0.3 (NCSS), resulting in an enrollment of 110 participants (55 allocated to the experimental group and 55 to the control group). Missing primary outcome data were assumed to be missing at random and imputed by multiple imputation by chained equations. Missing data were not imputed for secondary outcome analyses.

Continuous variables are presented as mean (SD) or median (IQR), as appropriate. Categorical variables were described as frequency and proportion. For continuous variables, independent samples *t* tests or Mann-Whitney tests were applied, depending on the data distribution. χ^2^ Tests were performed for categorical variables, and Kruskal-Wallis tests for ordinal variables. UC rates 20 weeks after randomization were calculated using Kaplan-Meier curves, and the log-rank test was used for the curve comparison. A post hoc modified Poisson regression analysis was conducted in the intention-to-treat population to adjust for nerve-sparing techniques and diabetes status for the primary outcome. Statistical analyses were conducted using SPSS software, version 29.0 (IBM Corporation) and R software, version 4.4.1 (R Program for Statistical Computing). All tests were 2-tailed, and *P* < .05 was considered significant. Both primary and secondary outcomes were analyzed in accordance with the intention-to-treat principle. We also conducted exploratory post hoc subgroup analyses based on the baseline information of the patients to identify potential subgroups that may benefit more from electroacupuncture stimulation.

## Results

### Baseline Information

**Figure 2.**
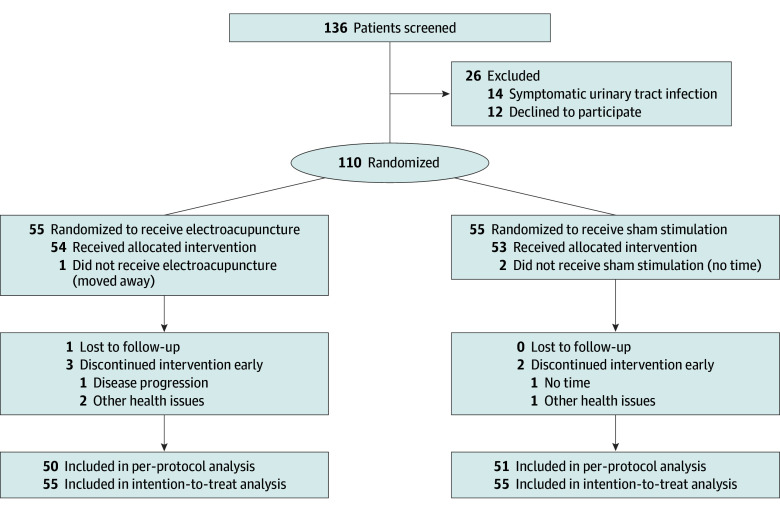
Study Flowchart

A total of 110 eligible and consenting men (median age, 69 [IQR, 67-72] years) were randomly allocated to either the electroacupuncture group or the sham stimulation group, with 55 patients in each group, and all were included in the intention-to-treat analyses (Figure 2). After randomization, 3 patients (1 in the electroacupuncture group and 2 in the sham stimulation group) did not receive the allocated intervention. Additionally, 1 patient in the electroacupuncture group was lost to follow-up, and a total of 5 patients (3 in the electroacupuncture group and 2 in the sham stimulation group) discontinued the intervention early. Among the 110 men included in the intention-to-treat analysis, 1 had a missing primary outcome.

As presented in Table 1, the overall workflow timelines were comparable between the 2 groups. The median time from RP to enrollment was 31 (IQR, 30-33) days for the sham stimulation group and 31 (IQR, 29-33) days for the electroacupuncture group. Similarly, the median time from randomization to intervention initiation was 3 (IQR, 2-4) days in the sham stimulation group and 3 (IQR, 1-4) days in the electroacupuncture group. The follow-up duration was 20 weeks, with no instances of patient crossover between the treatment groups.

**Table 1.** Participant Baseline Characteristics

Characteristic	Participants, No. (%)		
	All (N = 110)	Sham stimulation (n = 55)	Electroacupuncture (n = 55)
Age, median (IQR), y	69 (67-72)	69 (67-72)	69 (67-72)
BMI, mean (SD)	24.35 (2.91)	24.67 (3.24)	24.02 (2.53)
Educational attainment			
Primary school	10 (9.1)	5 (9.1)	5 (9.1)
Junior high	32 (29.1)	14 (25.5)	18 (32.7)
High school or technical school	37 (33.6)	22 (40.0)	15 (27.3)
College or above	31 (28.2)	14 (25.5)	17 (30.9)
Smoking	38 (34.5)	18 (32.7)	20 (36.4)
Alcohol consumption	17 (15.5)	9 (16.4)	8 (14.5)
Hypertension	66 (60.0)	34 (61.8)	32 (58.2)
Diabetes	17 (15.5)	11 (20.0)	6 (10.9)
Preoperative PSA level, median (IQR), ng/ml	6.77 (4.99-10.63)	6.84 (4.47-11.40)	6.75 (5.10-9.80)
Biopsy ISUP grade group			
1	20 (18.2)	7 (12.7)	13 (23.6)
2	60 (54.5)	31 (56.4)	29 (52.7)
3	20 (18.2)	11 (20.0)	9 (16.4)
4 or 5	10 (9.1)	6 (10.9)	4 (7.3)
Clinical T stage			
T1c	10 (9.1)	5 (9.1)	5 (9.1)
T2a	51 (46.4)	25 (45.5)	26 (47.3)
T2b	20 (18.2)	11 (20.0)	9 (16.4)
T2c	29 (26.4)	14 (25.5)	15 (27.3)
PLND	28 (25.5)	15 (27.3)	13 (23.6)
Nerve-sparing technique			
Unilateral	23 (20.9)	8 (14.5)	15 (27.3)
Bilateral	42 (38.2)	23 (41.8)	19 (34.5)
ECOG status			
0	102 (92.7)	51 (92.7)	51 (92.7)
1	8 (7.3)	4 (7.3)	4 (7.3)
Baseline EPIC-CP UI score, median (IQR)^a^	9 (8-10)	9 (8-10)	9 (8-10)
Baseline 24-h pad weight, median (IQR), g	500 (337.5-712.5)	500 (350-800)	500 (300-700)
Baseline 24-h pad number, median (IQR)	3 (2-3)	3 (2-3)	2 (2-3)
Workflow times, median (IQR), d			
RP to enrollment	31 (29-33)	31 (30-33)	31 (29-33)
Randomization to intervention	3 (1-4)	3 (2-4)	2 (1-4)

Abbreviations: BMI, body mass index (calculated as weight in kilograms divided by height in meters squared); ECOG, Eastern Cooperative Oncology Group; EPIC-CP, Expanded Prostate Cancer Index Composite for Clinical Practice; ISUP, International Society for Urological Pathology; PLND, pelvic lymph node dissection; RP, radical prostatectomy; PSA, prostate-specific antigen; UI, urinary incontinence.

SI conversion factor: To convert PSA values to micrograms per liter, multiply by 1.

^a^ Scores range from 0 to 12, with higher scores indicating greater bother or severity of symptoms related to urinary incontinence.

### Primary Outcome

The primary outcome of UC rate after the 6-week treatment period was significantly higher in the electroacupuncture group (24 of 55 [43.6%]) compared with the sham stimulation group (12 of 55 [21.8%]), with a relative risk of 2.00 (95% CI, 1.12-3.59; *P* = .02) (Table 2). The result remained significant in the post hoc sensitivity analysis adjusted for nerve-sparing techniques and diabetes status (adjusted relative risk, 1.96; 95% CI, 1.09-3.52; *P* = .03) (eTable 1 in Supplement 2).

**Table 2.** Primary and Secondary Outcomes

Variable	Participants		Comparison (95% CI)	*P* value
	Sham stimulation (n = 55)	Electroacupuncture (n = 55)		
Primary outcome, No./total No. (%)				
UC rate	12/55 (21.8)	24/55 (43.6)	2.00 (1.12 to 3.59)^a^	.02^b^
Secondary outcome, median (IQR)				
Changes in the amount of 24-h urine leakage, g	−200 (−300 to −100)	−320 (−483 to −198)	−140 (−200 to −50)^c^	<.001^d^
Changes in mean No. of pads used	−1.0 (−1.0 to 0.0)	−1.0 (−2.0 to −0.0)	−0.5 (−1.0 to 0.0)^c^	.02^d^
Changes in EPIC-CP UI score	−2 (−3 to −1)	−3 (−5 to −2)	−2 (−1 to 0)^c^	.01^d^

Abbreviations: EPIC-CP, expanded prostate cancer index composite for clinical practice; UC, urinary continence; UI, urinary incontinence.

^a^ Described as relative risk (95% CI).

^b^ Calculated using Pearson χ^2^ test.

^c^ Described as difference (95% CI).

^d^ Calculated using Mann-Whitney test.

### Secondary Outcomes

**Figure 3.**
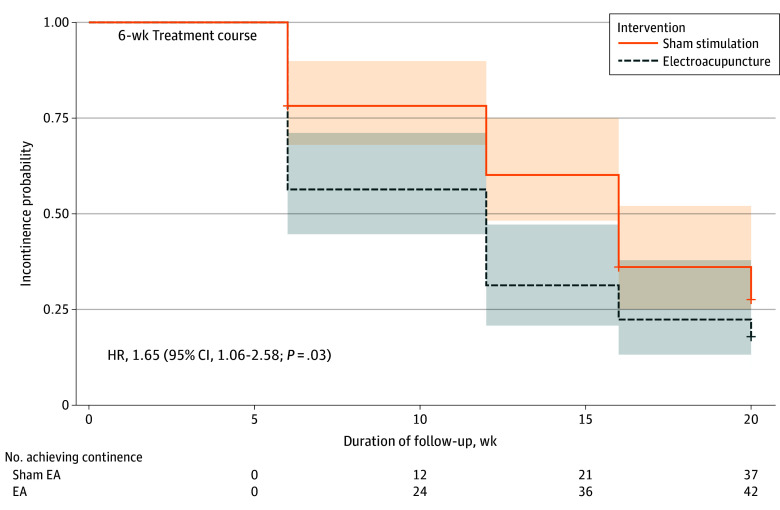
Kaplan-Meier Curves Showing the Long-Term Urinary Continence Recovery in Patients Receiving Electroacupuncture and Sham Stimulation EA indicates electroacupuncture; HR, hazard ratio. The shading surrounding the survival curves represents the 95% CIs.

The electroacupuncture group demonstrated a more pronounced reduction in the amount of 24-hour urine leakage, with a median change of −320 (IQR, −483 to −198) g compared with −200 (IQR, −300 to −100) g in the sham stimulation group, representing a significant difference of −140 (95% CI, −200 to −50) g (*P* < .001). Similarly, the electroacupuncture group showed a greater reduction in the mean number pads used daily, with a median change of −1.0 (IQR, −2.0 to 0) compared with −1.0 (IQR, −1.0 to 0) in the sham stimulation group (difference, −0.5; 95% CI, −1.0 to 0; *P* = .02). Prostate-related symptoms assessed by the EPIC-CP UI score also improved more in the electroacupuncture group, with a median change of −3 (IQR, −5 to −2) compared with −2 (IQR, −3 to −1) in the sham stimulation group (difference, −2; 95% CI, −1 to 0; *P* = .01). Last, Kaplan-Meier analysis demonstrated significantly shorter time to UC recovery in the electroacupuncture group during the 20-week follow-up period (hazard ratio, 1.65; 95% CI, 1.06-2.58; log-rank *P* = .03), which is depicted in Figure 3. The survival analysis met the proportional hazards assumption via the Schoenfeld residual method (*P* = .08). By week 20, cumulative UC rates included 42 of 55 patients (76.4%) in the electroacupuncture group vs 37 of 55 (67.3%) in the sham stimulation group (*P* = .29).

### Exploratory Post Hoc Subgroup Analyses

Exploratory post hoc subgroup analyses were conducted based on patients’ baseline characteristics. The benefits of electroacupuncture were not significantly different across most subgroups, except for the subgroup-stratified bay nerve–sparing involvement (eFigure in Supplement 2). Electroacupuncture efficacy was greater in patients with unilateral (odds ratio [OR], 0.12; 95% CI, 0.01-0.98) or bilateral (OR, 0.14; 95% CI, 0.03-0.61) nerve-sparing technique during RP compared with those with none (OR, 1.60; 95% CI, 0.43-5.96; *P* for interaction = 0.02).

### Adverse Events

Adverse events related to the treatment were documented and are presented in eTable 2 in Supplement 2. Overall, the incidence of adverse events was low, with only 1 event in 51 patients (1.8%) in the sham stimulation group and 2 events in 50 patients (4.0%) in the electroacupuncture group. No severe adverse events were reported. Local muscle spasms occurred in 1 participant in the sham stimulation group. Pain induced by needle insertion and subcutaneous hemorrhage were each reported in 1 participant in the electroacupuncture group.

## Discussion

This randomized clinical trial demonstrates that electroacupuncture significantly improves UC recovery in men following RP. The electroacupuncture group achieved a UC rate of 43.6% at 6 weeks, doubling the rate observed in the sham stimulation group (21.8%), with a relative risk of 2.00 (95% CI, 1.12-3.59). Notably, our UC criteria (0-1 pad per day) align with the safety pad concept increasingly adopted in recent studies,^5^ which balances clinical relevance with patient-reported convenience. While the International Continence Society defines UI as the complaint of any involuntary leakage of urine,^31^ clinical practice often tolerates minimal pad use for psychological reassurance. A prior reverse systematic review (including 51 436 patients) found no significant difference in UC rates between definitions of no pad and safety pad within 12 postoperative months.^30^ This supports the clinical validity of our end point selection.

The superiority of electroacupuncture over sham stimulation aligns with previous evidence on neuromodulation therapies for SUI. The observed 24-hour urine leakage reduction (−320 vs −200 g) and pad use reduction (difference, −0.5; *P* = .02) provide dual validation of treatment efficacy. Kaplan-Meier analysis demonstrated a significantly shorter time to UC with electroacupuncture compared with sham stimulation (hazard ratio, 1.65; 95% CI, 1.06-2.58; log-rank *P* = .03). Although this difference reflected a clinically meaningful benefit, the gap between the 2 groups gradually narrowed over time, suggesting that electroacupuncture may primarily accelerate the recovery from UI through neuromodulation rather than fundamentally altering the long-term trajectory. This is consistent with the natural history of UI after RP, which typically improves spontaneously within 6 to 12 months.^5,32^ However, the early achievement of UC in the electroacupuncture group may alleviate the burden on patients during the critical recovery period, potentially mitigating psychological distress and improving quality of life.^33^ Our findings extend electroacupuncture’s utility beyond female SUI,^13,17^ supporting the hypothesis that electroacupuncture exerts therapeutic effects through similar mechanisms, likely by stimulating sacral nerve roots (S2-S4) via the Baliao acupoints (BL32-BL34).^17,19^ Our post hoc analysis also aligns with the neuromodulation hypothesis, which identified a significant interaction between electroacupuncture efficacy and nerve-sparing status (*P* = .02 for interaction). Patients who underwent unilateral (OR, 0.12; 95% CI, 0.01-0.98) or bilateral (OR, 0.14; 95% CI, 0.03-0.61) nerve-sparing technique derived substantially greater benefit from electroacupuncture compared with those who did not (OR, 1.60; 95% CI, 0.43-5.96). Previous studies have suggested that electroacupuncture stimulation of acupoints such as BL32 and BL33 may improve UC by modulating sacral nerve activity to enhance intrinsic sphincter function and activating γ-aminobutyric acid–related neurons to inhibit bladder overactivity.^34,35,36^ During electroacupuncture sessions, some of the patients frequently reported sphincteric contractions and sensations. However, previous studies on electroacupuncture effects using functional magnetic resonance imaging have also shown changes in activities of brain regions.^37,38^ Therefore, we hypothesize that the observed therapeutic effects of electroacupuncture in our study may be attributed to a combination of both peripheral and central neuromodulating pathways. The bilateral application of electroacupuncture at these points may facilitate reinnervation of damaged sphincteric muscles and improve urethral closure pressure, as evidenced by both functional metrics (43.6% UC rate) and objective leakage parameters (reduction of −320 g). The EPIC-CP instrument proved particularly advantageous for capturing nuanced health-related quality of life, with its UI domain minimally important difference of 1.0.^39^ Our electroacupuncture group’s score reduction (−3) significantly exceeded this threshold, indicating clinically meaningful improvement. The simplicity of EPIC-CP UI score’s range from 0 to 12 enabled efficient integration into follow-up visits without disrupting clinical workflow, a critical factor for patient-reported outcome adoption in clinical settings.

Clinically, the 43.6% UC rate at 6 weeks in our cohort, which was composed exclusively of patients with persistent UI at 1 month post RP, compares favorably to the 37.5% UC rate reported in historical cohorts evaluating PFMT alone at 3 months.^12^ This difference likely reflects stricter patient selection in our trial: whereas prior studies included all patients regardless of early UC status (thereby inflating natural recovery rates),^12,27^ our cohort specifically targeted men with delayed recovery (baseline UI at 4-6 postoperative weeks). This approach allowed us to focus on a more homogeneous and clinically relevant patient population, thereby providing a clearer assessment of the therapeutic potential of electroacupuncture in this challenging group. The low rate of adverse events (4.0% in electroacupuncture and 1.8% in sham stimulation) reinforces electroacupuncture’s safety profile, consistent with prior studies.^17,19^

Compared with previous studies, the present study has made improvements by using electroacupuncture as the intervention, integrating modern neuromodulation theories, instead of traditional methods relying solely on TCM theories.^21,22^ Moreover, the sham stimulation control group design helps to more accurately assess the therapeutic effects of electroacupuncture and avoids biases caused by the absence of a control.

### Limitations

This study has some limitations. First, the single-center design may limit generalizability. Second, while we standardized PFMT protocols, compliance variations could introduce bias. Third, the post hoc nature of the subgroup analyses restricted the interpretation of the correlation of nerve-sparing technique and electroacupuncture stimulation. Fourth, we did not include the 3-day bladder diary, which could have provided more detailed information on UI patterns. This was a compromise made to minimize the burden on participants and ensure high adherence with the study protocol. Future trials with multicenter cohorts, prespecified hypotheses, and objective urodynamic measures are needed to further validate electroacupuncture’s efficacy on postprostatectomy UI and its potential mechanisms.

## Conclusions

In this randomized clinical trial of 110 men with early UI after RP, electroacupuncture significantly improved UC compared with sham stimulation after the 6-week course with minimal adverse events. By using a clinically pragmatic UC definition and leveraging EPIC-CP’s efficiency, our approach bridges research rigor with clinical applicability. This trial provides robust evidence that electroacupuncture may enhance early UC recovery after RP but may not change the long-term continence outcomes.

## Notes

Supplement 1.
Trial Protocol

Supplement 2. **eTable 1.** Sensitivity Analysis Adjusting for Diabetes and Nerve-Sparing Status **eTable 2.** Adverse Events Related to Treatment **eFigure.** Forest Plot Showing the Exploratory Post Hoc Subgroup Analyses

Supplement 3.
Data Sharing Statement
