# Supplementary material for: Electroacupuncture in Patients With Early Urinary Incontinence After Radical Prostatectomy: A Randomized Clinical Trial
Source: JAMA Netw Open. 2025 Sep 30;8(9):e2534491. doi: 10.1001/jamanetworkopen.2025.34491 (PMC12485641; doi:10.1001/jamanetworkopen.2025.34491)
Supplement: Supplement 1. — Trial Protocol [file jamanetwopen-e2534491-s001.pdf]

Effect of Electroacupuncture on Early Urinary Continence After Radical Prostatectomy: A Prospective, Single-Center, Randomized, Single-Blinded Clinical Trial

Trial Registration

This trial was registered on ClinicalTrials.gov, number NCT04901130.

Protocol Version

Version 2.0 Date: 21-Dec-2021.

Funding

This study was supported by grants from the National Natural Science Foundation of China (ID: 82172639), the Project of Invigorating Health Care through Science, Technology and Education, Jiangsu Provincial Key Medical Discipline (Laboratory) (ZDXKB2016014), Nanjing Drum Tower Hospital Clinical Research Special Funding Program (2023-LCYJ-MS-15), and the Sino-German Mobility Programme (M-0670), Jiangsu Province Capability Improvement Project through Science, Technology and Education, Jiangsu Provincial Medical Key Discipline (Laboratory) Cultivation Unit (JSDW202221).

1. Roles and Responsibilities

1.1 Protocol Contributors

1） Principal Investigators:

• Hongqian Guo, MD, PhD (Department of Urology; Nanjing Drum Tower Hospital, Affiliated Hospital of Medical School, Nanjing University, Institute of Urology, Nanjing University): Corresponding author, oversees trial design, protocol implementation, final data interpretation and obtained funding.

• Xufeng Qiu, MD, PhD (Department of Urology, Nanjing Drum Tower Hospital, Affiliated Hospital of Medical School, Nanjing University; Institute of Urology, Nanjing University): Corresponding author, concepts the trial, and manages participant recruitment, ethical compliance, obtained funding and data analysis.

2）Co-investigators:

• Jiahui Niu (Department of Urology, Nanjing Drum Tower Hospital, Affiliated Hospital of Medical School, Nanjing University; Institute of Urology, Nanjing University): Collects data, performs data analysis, and draft writing.

• Yang Wang and Yujuan Wang (Department of Traditional Chinese Medicine, Nanjing Drum Tower Hospital, Affiliated Hospital of Medical School, Nanjing University): Intervention delivery.

• Jingyan Shi, PhD (Department of Urology, Nanjing Drum Tower Hospital, Affiliated Hospital of Medical School, Nanjing University; Institute of Urology, Nanjing University): Executes randomization, and ensures binding integrity.

• Jing Liang and Xiaozhi Zhao (Department of Urology, Nanjing Drum Tower Hospital, Affiliated Hospital of Medical School, Nanjing University): Supervises outcome assessments.

• Xiaoyu Lyu and Mengxia Chen (Department of Urology, Nanjing Drum Tower Hospital, Affiliated Hospital of Medical School, Nanjing University; Institute of Urology, Nanjing University): Conduct telephone follow-up and monitor adverse events.

1.2 Steering Committee

1）Role: Provides overarching oversight, reviews protocol amendments, and resolves operational challenges.

2）Members:

• Hongqian Guo (Chair)

• Xuefeng Qiu

• Tianshu Xu (Traditional Chinese Medicine Specialist)

• Biyun Xu (Independent statistician, blinded to allocation)

1.3 Date and Safety Monitoring Board (DSMB)

An independent DSMB will oversee participant safety, review interim analyses and ensure data integrity. The composing members are as follows:

Linfeng Xu, MD: Chair of the DSMB, unblinded to group allocation but excluded from outcome assessment and statistical analysis.

Junlong Zhuang, MD, PhD: Voting member of the DSMB, providing clinical expertise and input on participant safety.

Wenli Diao, PhD: Voting member of the DSMB, contributing to the evaluation of adverse events and safety monitoring.

Ru Fan: Voting member of the DSMB, providing statistical expertise and analysis support for interim data reviews.

2.Introduction

2.1 Background and Rationale

Radical prostatectomy (RP) is a primary treatment for localized prostate cancer (PCa), offering high cancer-specific- survival (CSS) at 10-year follow-up[1], but is associated with early post-prostatectomy urinary incontinence (UI) in 25-86% patients[2,3]. UI severely impacts patients' quality of life[4,5], and existing therapies like pelvic muscle training (PFMT) and artificial urinary sphincters (AUS) exhibit limited efficacy or invasiveness[6-8]. Electroacupuncture (EA) combines neuromodulation and Traditional Chinese Medicine (TCM) theory, demonstrating promise in female stress UI but lacking evidence in males[9,10]. A rigorous evaluation of EA's efficacy and safety in men, using a standardized sham-controlled design, is critical to inform clinical practice. A sham acupuncture protocol was selected to isolate EA's specific effects from nonspecific factors (e.g., placebo, patient-provider interaction). Flat-tip needles applied at sham acupoints mimic EA's tactile sensation without active stimulation, ensuring blinding validity. This approach aligns with recommendations from the Standards for Reporting Interventions in Clinical Trials of Acupuncture (STRICTA) guidelines[11].

2.2 Objective

This prospective, single-blinded, randomized clinical trial was designed to assess the effectiveness of EA for the treatment of UI after RP.

2.3 Trial Design

This study is a prospective, single-center, randomized, single-blinded, sham-controlled clinical trial designed to evaluate EA's efficacy and safety in men with post-RP UI. Participants are randomized (1:1) to receive either EA or sham EA for 6 weeks, with follow-up extending to 20 weeks post-randomization. Blinding is maintained for participants and outcome assessors, while acupuncturists are unblinded to ensure protocol adherence. The trial adheres to Consolidated Standards of Reporting Trials (CONSORT) guidelines to ensure methodological rigor and transparency[12].

3. Methods: Participants, Interventions and outcomes

3.1 Study Setting

This single-center trial is conducted at the Department of Urology, Nanjing Drum Tower Hospital, Affiliated Hospital of Medical School, Nanjing University, China, a tertiary care academic medical center with extensive experience in the treatment of prostate cancer and urinary incontinence. Data collection will be limited to this single center to ensure consistency in study procedures and interventions. All interventions, assessments, and follow-ups are performed in a standardized clinical research unit equipped with dedicated acupuncture rooms and urological rehabilitation facilities.

3.2 Eligibility Criteria

1) Inclusion Criteria

(a) Localized prostate cancer (stage: cT1-cT2, N0, M0);

(b) 4-6 weeks after robot-assisted RP (RARP);

(c) Mean 24-h pad number $\geq$ 2;

(d) Prostate-specific antigen (PSA) test below cured level 1 month after surgery;

(e) Eastern Cooperative Oncology Group (ECOG) performance status of 0-1

2) Exclusion Criteria

(a) Any prior neoadjuvant hormonal therapy;

(b) Any prior prostatic surgeries;

(c) Any prior history of operation on pelvis;

(d) History of incontinence before RP.

3.3 Interventions

The intervention report adheres to the Standards for Reporting Interventions in Clinical Trials of Acupuncture (STRICTA) guidelines[11].

1) Experimental Group

Based on the principles of TCM acupuncture theory and neuroanatomical structures, the intervention in this study involves EA at specific acupoints. The selected dacupuncture sites were bilateral Ciliao (BL32), Zhongliao (BL33) and Xialiao (BL34) acupoints on the Bladder Meridian[13], which are closely associated with the sacral micturition center and are located in the second, third and fourth sacral foramina, respectively (Figure 1)[14,15].

Patients in the experimental group receive EA stimulation at these acupoints. After skin disinfection, sterile adhesive pads were placed on predetermined acupoints. Single-use, sterile acupuncture needles with a diameter of 0.30 mm and a length of 75mm will be used. The needles are inserted through the adhesive pads at specific angles: the angles between the needle shaft and the skin surface range from 45° to 65°, and the optimal angles between the needle shaft and the posterior midline of the body for needle insertion are BL34 (20-40)°, BL32 and BL33 (10-25)°. The optimal depths for electrostimulation vary from cranial to caudal, with depths of 35-55mm for BL32, 25-45mm for BL33, and 20-40 mm for BL 34. Following needle insertion, small, equal manipulations of twirling, lifting, and thrusting were performed on all needles to reach de qi, a complex sensation characterized by soreness, numbness, swelling, and heaviness.After de qi is achieved, the bilateral BL32 (anode) and BL33 (cathode) are connected to the SDZ-V type electronic acupuncture therapy device (Suzhou Medical Supplies Factory Co., Ltd.). A sparse-dense wave, with a frequency of 2Hz/15Hz, will be applied, and the current intensity was adjusted to the maximum tolerable level for the patient, with the needles retained for 30 minutes. Participants receive 3 treatment sessions per week (ideally every other day) for 6 consecutive weeks, 18 sessions in total.

2) Control Group

Participants in the control group undergo identical preparation procedures, but utilize single-use, custom-made flat-tip acupuncture needles (0.30 mm diameter, 75 mm length) to penetrate the adhesive pads without skin puncture. The sham EA points are offset one cun (approximately 20 mm) laterally from the experimental group's points. No de qi manipulation will be performed, and the duration and frequency of current output will be the same as in the experimental group. The treatment course and intervals for the control group are consistent with those of the experimental group.

All participants are suggested to undergo PFMT as part of standardized postoperative care. In accordance with our institutional protocol, PFMT will be initiated after catheter removal, as recommended by AUA/SUFU guideline[4]. Participants performed three sets of pelvic floor

muscle exercises daily, with each set consisting of 10 contractions held for 10 seconds each, with a 10-second rest between contractions[16,17].

3) Criteria for Discontinuing or Modifying Allocated Interventions

(a) Participant withdrawal of consent
(b) Severe adverse events (e.g. persistent pain, infection) requiring discontinuation
(c) Disease progression necessitating alternative therapies.

4) Strategies to Improve Adherence

(a) Reminder calls/notifications before each session.
(b) Flexible scheduling to accommodate participant availability
(c) Adherence monitoring via session attendance logs reviewed weekly by the study coordinator.

5) Permitted Concomitant Care and Prohibited Interventions

Analgesics (e.g. nonsteroidal anti-inflammatory drugs) are allowed for unrelated conditions but documented. Additional acupuncture, neuromodulation therapies, or surgical interventions for UI during the trial period is prohibited.

3.4 Outcomes

1) Primary Outcome

UC (define as the use of 0 to 1 pad per day) rate after the 6-week treatment period.

2) Secondary Outcomes

(a) Changes in the amount of 24-h urine leakage at the end of 6-week treatment period compared with baseline.
(b) Changes in the mean number of pads used daily at the end of 6-week treatment period compared with baseline.
(c) Prostate-related symptoms assessed by changes in the Expanded Prostate Cancer Index Composite for Clinical Practice (EPIC-CP) UI score at the end of 6-week treatment period compared with baseline.
(d) Time to UC recovery in weeks.

3.5 Participant Timeline

A detailed schedule of enrollment, randomization, intervention and follow-up is provided in Figure 2. The study involves a 6-week intervention period accompanied with a 20-week follow-up period. Participants will be assessed at baseline, at the end of the intervention period, and at 12, 16, and 20weeks post-randomization.

3.6 Allocation and Blinding

The randomization sequence was generated by an independent statistician using a computerized random number generator (R version 3.6.0). The sequence was designed to ensure a 1:1 allocation ratio between the EA group and the sham EA group. The allocation sequence was concealed using sequentially, numbered, opaque, sealed envelopes. After baseline assessments, a research assistant (uninvolved in recruitment, intervention delivery, or outcome evaluation) opens the next sequentially numberer envelope in the presence of the participant to reveal group assignment. Participants, outcome assessors, and data analysts were blinded to the intervention allocation. The acupuncturists were aware of the group assignment due to the nature of the intervention. Unblinding was permitted only in emergencies (e.g. severe adverse events requiring knowledge of intervention for clinical management).

3.7 Date Collection and Management

Data collection will be conducted using standardized case report forms (CRFs). Baseline data include demographic information (age, body mass index [BMI]), medical history (history of hypertension, history of diabetes), unhealthy habits (smoking history, alcohol consumption), oncological features (preoperative PSA, biopsy International Society for Urological Pathology [ISUP] grade group, clinical T stage, Eastern Cooperative Oncology Group [ECOG] status, involvement of pelvic lymph node dissection [PLND], and nerve-sparing status), and initial UC status (number of pads used per day, 24-h pad weight, Expanded Prostate Cancer Index Composite for Clinical Practice [EPIC-CP] UI score). Outcome data will be collected after the 6-week intervention period, and at 12, 16, and 20weeks post-randomization.

Data management is overseen by an independent DSMB composed of experienced clinical research coordinators and statisticians. The DSMB will ensure the accuracy, completeness, and confidentiality of all study data by conducting monthly comprehensive data checks to identify systematic errors or problems in data entry. Manual checks will be performed to identify more complex and less common errors. Any discrepancies or missing data will be flagged and resolved through communication with the study sites. Only authorized personnel will have access to the data and all data will be anonymized to protect participant confidentiality. Any requests for data access will be reviewed and approved by the DSMB.

3.8 Statistical Analysis

1) Sample Size Calculation

Based on preliminary data from our institution, the UC rate was 20% for those who performed PFMT alone 3 months postoperatively, 48% for those who underwent 6-week EA treatment. Assuming a two-tailed significant level of 0.05 and 80% power, a total of 110 participants (55 per group) will be required. This calculation accounts for a 10% dropout rate.

2) Data Analysis Plan

All analyses will be performed using the intention-to-treat (ITT) principle, including all randomized participants. Continuous variables are summarized using means with standard deviations (SD) or medians with interquartile ranges (IQR), as appropriate. Categorical variables are described using frequencies and percentages. For variables, Student t-tests or Mann-Whitney U tests are applied, depending on the data distribution. Chi-square tests are used for categorical variables, and Kruskal-Wallis tests are used for ordinal variables. Statistical analyses are conducted using SPSS software, version 21.0 or higher (IBM, USA) and R software (version 3.6.0 or higher).

4. Ethics and Dissemination

4.1 Ethical approval

This clinical trial was approved by the Review Board of Nanjing Drum Tower Hospital (2021-083-02) and registered on ClinicalTrials.gov (NCT04972669). The study adheres to the principles of the Declaration of Helsinki and Good Clinical Practice guidelines. All participants should provide written informed consent prior to enrollment in the study.

4.2 Protocol Amendments

Any modifications to the protocol will be documented and submitted to the ethics committee for approval. The trial participants, trial registries and relevant parties will be promptly informed of any significant protocol amendments.

4.3 Consent

Eligible participants receive detailed oral and written explanations of the trial's purpose, procedures, risks and benefits. Signed informed consent forms are obtained by trained research coordinators before enrollment. Participants who are unable to provide consent due to cognitive impairment or other reasons are excluded to ensure voluntary participation. Participants may withdraw consent at any time without affecting their clinical care.

4.4 Dissemination Policy

The results of the trial will be disseminated through peer-reviewed publications, conference presentations, and other relevant scientific forums. The authors will adhere to the guidelines for reporting clinical trials, including the CONSORT and Standard Protocol Items: Recommendations for Interventional Trials (SPIRIT) statement to ensure transparent an accurate reporting of the study findings[12,18]. The data collected for the present study, including deidentified participant data will be made available to others. These data can be made available following communication with Dr. Xuefeng Qiu (Xuefeng_qiu@nju.edu.cn). Before these data are shared (with professional colleagues or other investigators), the request will be

reviewed by Dr. Xuefeng Qiu. All proposals must be approved by Dr. Xuefeng Qiu, with a signed data access agreement.

Declaration of Interests

The principal investigators and all members of the research team declare no financial or other competing interests in relation to this trial. The funding sources have no role in the design and conduct of the study; collection, management, analysis, and interpretation of the data; preparation, review, or approval of the manuscript; or decision to submit the manuscript for publication.

1 Cornford, P. *et al.* EAU-EANM-ESTRO-ESUR-ISUP-SIOG Guidelines on Prostate Cancer—2024 Update. Part I: Screening, Diagnosis, and Local Treatment with Curative Intent. *European Urology* **86**, 148-163, doi:10.1016/j.eururo.2024.03.027 (2024).

2 Ficarra, V. *et al.* Systematic Review and Meta-analysis of Studies Reporting Urinary Continence Recovery After Robot-assisted Radical Prostatectomy. *European Urology* **62**, 405-417, doi:10.1016/j.eururo.2012.05.045 (2012).

3 Dalela, D. *et al.* A Pragmatic Randomized Controlled Trial Examining the Impact of the Retzius-sparing Approach on Early Urinary Continence Recovery After Robot-assisted Radical Prostatectomy. *European Urology* **72**, 677-685, doi:10.1016/j.eururo.2017.04.029 (2017).

4 Sandhu, J. S. *et al.* Incontinence after Prostate Treatment: AUA/SUFU Guideline. *Journal of Urology* **202**, 369-378, doi:10.1097/ju.0000000000000314 (2019).

5 Li, Y. *et al.* Recent Advances in Diagnosing and Treating Post-Prostatectomy Urinary Incontinence. *Annals of Surgical Oncology* **31**, 8444-8459, doi:10.1245/s10434-024-16110-1 (2024).

6 Centemero, A. *et al.* Preoperative Pelvic Floor Muscle Exercise for Early Continence After Radical Prostatectomy: A Randomised Controlled Study. *European Urology* **57**, 1039-1044, doi:10.1016/j.eururo.2010.02.028 (2010).

7 Canning, A. *et al.* A systematic review of treatment options for post-prostatectomy incontinence. *World Journal of Urology* **40**, 2617-2626, doi:10.1007/s00345-022-04146-5 (2022).

8 Munier, P. *et al.* What if artificial urinary sphincter is not possible? Feasibility and effectiveness of ProACT for patients with persistent stress urinary incontinence after radical prostatectomy treated by sling. *Neurourology and Urodynamics* **39**, 1417-1422, doi:10.1002/nau.24355 (2020).

9 Liu, Z. *et al.* Effect of Electroacupuncture on Urinary Leakage Among Women With Stress Urinary Incontinence. *Jama* **317**, doi:10.1001/jama.2017.7220 (2017).

10 Tang, K. *et al.* Effect of Electroacupuncture Added to Pelvic Floor Muscle Training in Women with Stress Urinary Incontinence: A Randomized Clinical

Trial. *European Urology Focus* **9**, 352-360, doi:10.1016/j.euf.2022.10.005 (2023).
11 MacPherson, H. *et al.* Revised STandards for Reporting Interventions in Clinical Trials of Acupuncture (STRICTA): Extending the CONSORT Statement. *The Journal of Alternative and Complementary Medicine* **16**, ST-1-ST-14, doi:10.1089/acm.2010.1610 (2010).
12 Schulz, K. F., Altman, D. G., Moher, D. & Group, C. CONSORT 2010 statement: updated guidelines for reporting parallel group randomised trials. *BMJ* **340**, c332, doi:10.1136/bmj.c332 (2010).
13 Liu, Y., Liu, L. & Wang, X. Electroacupuncture at points Baliao and Huiyang (BL35) for post-stroke detrusor overactivity. *Neural Regen Res* **8**, 1663-1672, doi:10.3969/j.issn.1673-5374.2013.18.004 (2013).
14 Ozgoli, G., Sedigh Mobarakabadi, S., Heshmat, R., Alavi Majd, H. & Sheikhan, Z. Effect of LI4 and BL32 acupressure on labor pain and delivery outcome in the first stage of labor in primiparous women: A randomized controlled trial. *Complementary Therapies in Medicine* **29**, 175-180, doi:10.1016/j.ctim.2016.10.009 (2016).
15 Yang, L., Wang, Y., Mo, Q. & Liu, Z. A comparative study of electroacupuncture at Zhongliao (BL33) and other acupoints for overactive bladder symptoms. *Frontiers of Medicine* **11**, 129-136, doi:10.1007/s11684-016-0491-6 (2017).
16 Milios, J. E., Ackland, T. R. & Green, D. J. Pelvic floor muscle training in radical prostatectomy: a randomized controlled trial of the impacts on pelvic floor muscle function and urinary incontinence. *BMC Urology* **19**, doi:10.1186/s12894-019-0546-5 (2019).
17 Glazener, C. *et al.* Conservative treatment for urinary incontinence in Men After Prostate Surgery (MAPS): two parallel randomised controlled trials. *Health Technol Assess* **15**, 1-290, iii-iv, doi:10.3310/hta15240 (2011).
18 Chan, A. W. *et al.* SPIRIT 2013 statement: defining standard protocol items for clinical trials. *Ann Intern Med* **158**, 200-207, doi:10.7326/0003-4819-158-3-201302050-00583 (2013).

Figure 1. Acupoints' location for the electroacupuncture group and sham electroacupuncture group.

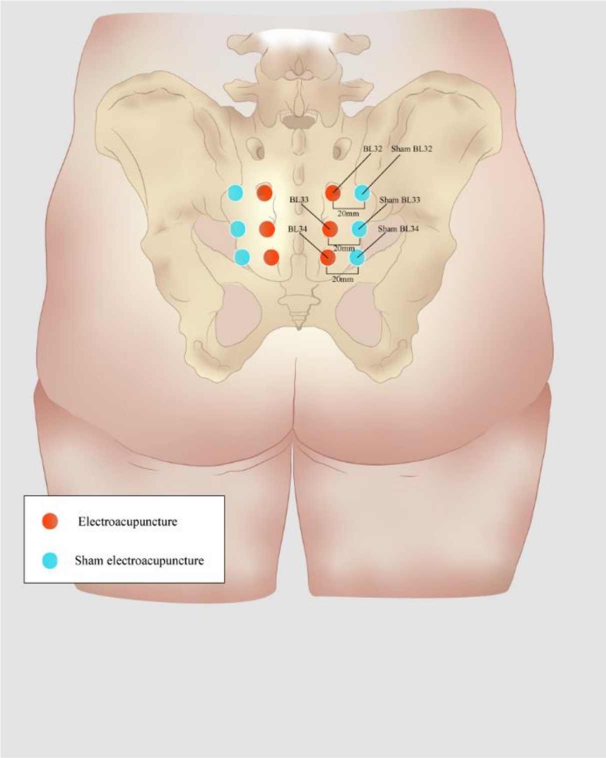

Figure 2. Enrollment Schedule

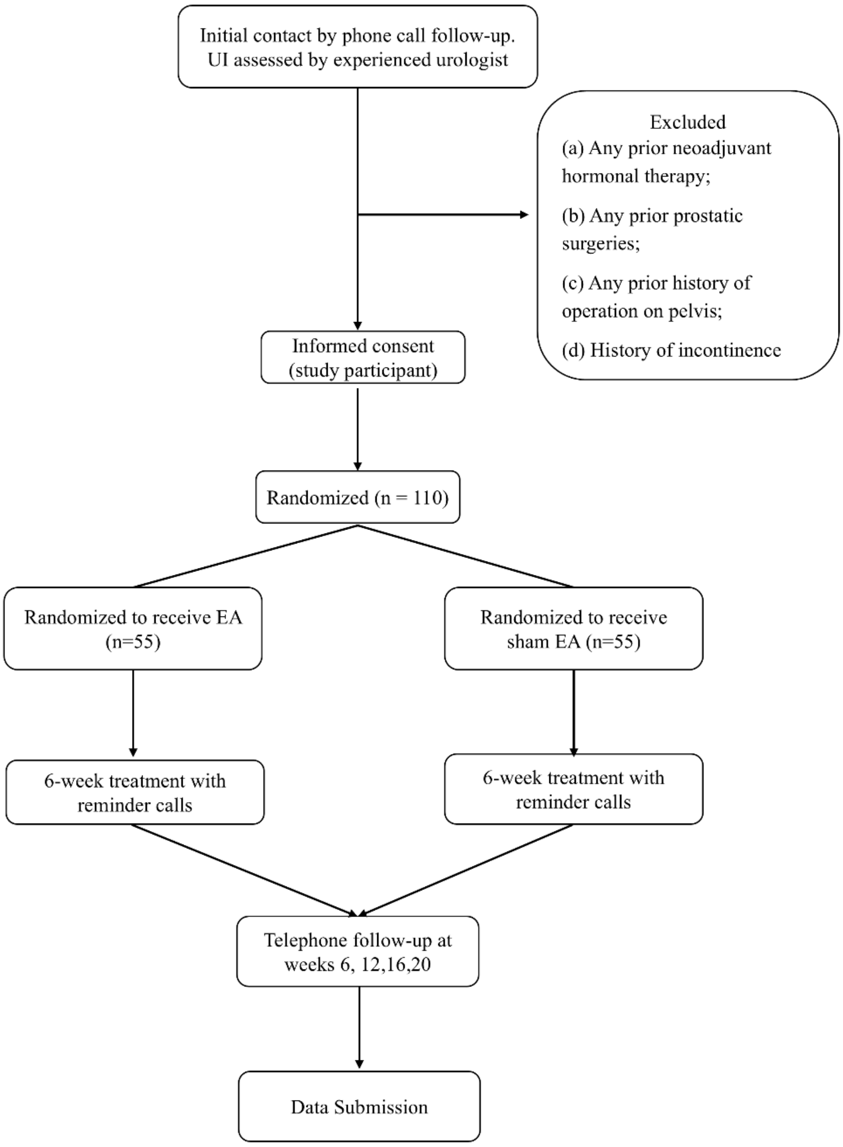

Abbreviations: UI, urinary incontinence; EA, electroacupuncture.
