# Supplementary material for: Electroacupuncture in Patients With Early Urinary Incontinence After Radical Prostatectomy: A Randomized Clinical Trial
Source: JAMA Netw Open. 2025 Sep 30;8(9):e2534491. doi: 10.1001/jamanetworkopen.2025.34491 (PMC12485641; doi:10.1001/jamanetworkopen.2025.34491)
Supplement: Supplement 2. — eTable 1. Sensitivity Analysis Adjusting for Diabetes and Nerve-Sparing Status eTable 2. Adverse Events Related to Treatment eFigure. Forest Plot Showing the Exploratory Post Hoc Subgroup Analyses [file jamanetwopen-e2534491-s002.pdf]

Supplementary Online Content

Niu J, Wang Y, Wang Y, et al. Electroacupuncture in patients with early urinary incontinence after radical prostatectomy: a randomized clinical trial. *JAMA Netw Open.* 2025;8(9):e2534491. doi:10.1001/jamanetworkopen.2025.34491

eTable 1. Sensitivity Analysis Adjusting for Diabetes and Nerve-Sparing Status

eTable 2. Adverse Events Related to Treatment

eFigure. Forest Plot Showing the Exploratory Post Hoc Subgroup Analyses

This supplementary material has been provided by the authors to give readers additional information about their work.

© 2025 Niu J et al. *JAMA Network Open*.

eTable 1. Sensitivity Analysis Adjusting for Diabetes and Nerve-Sparing Status

Outcome	Participants, No. (%)		ARR	P
	**Sham EA (n=55)**	**EA (n=55)**		
**Primary outcome**				
UC rate, n/N (%)	12/55 (21.8)	24/55 (43.6)	1.96 (1.09-3.52)	0.03 *

Abbreviations: EA, electroacupuncture; ARR, adjusted relative risk; CI, confidence interval; UC, urinary continence.

* calculated using modified Poisson regression.

eTable 2. Adverse Events Related to Treatment

Adverse events [a]	Participants, n (%)	
	Sham EA (n = 51) [b]	EA (n = 50) [b]
Overall	1 (1.8)	2 (4.0)
Severe adverse events	0	0
Local muscle spasms	1 (1.8)	0
Pain induced by needle insertion	0	1 (2.0)
Subcutaneous hemorrhage	0	1 (2.0)

Abbreviation: EA, electroacupuncture.

[a] Adverse events were analyzed in all participants who received treatment. Adverse events were counted by type rather than frequency in the same participant. Adverse events with different types occurring in a single participant were defined as independent adverse events. An adverse event with multiple occurrences in a single participant was defined as 1 adverse event.

[b] 5 participants in the EA group and 4 in the sham EA group did not receive treatment.

© 2025 Niu J et al. *JAMA Network Open*.

eFigure. Forest Plot Showing the Exploratory Post Hoc Subgroup Analyses

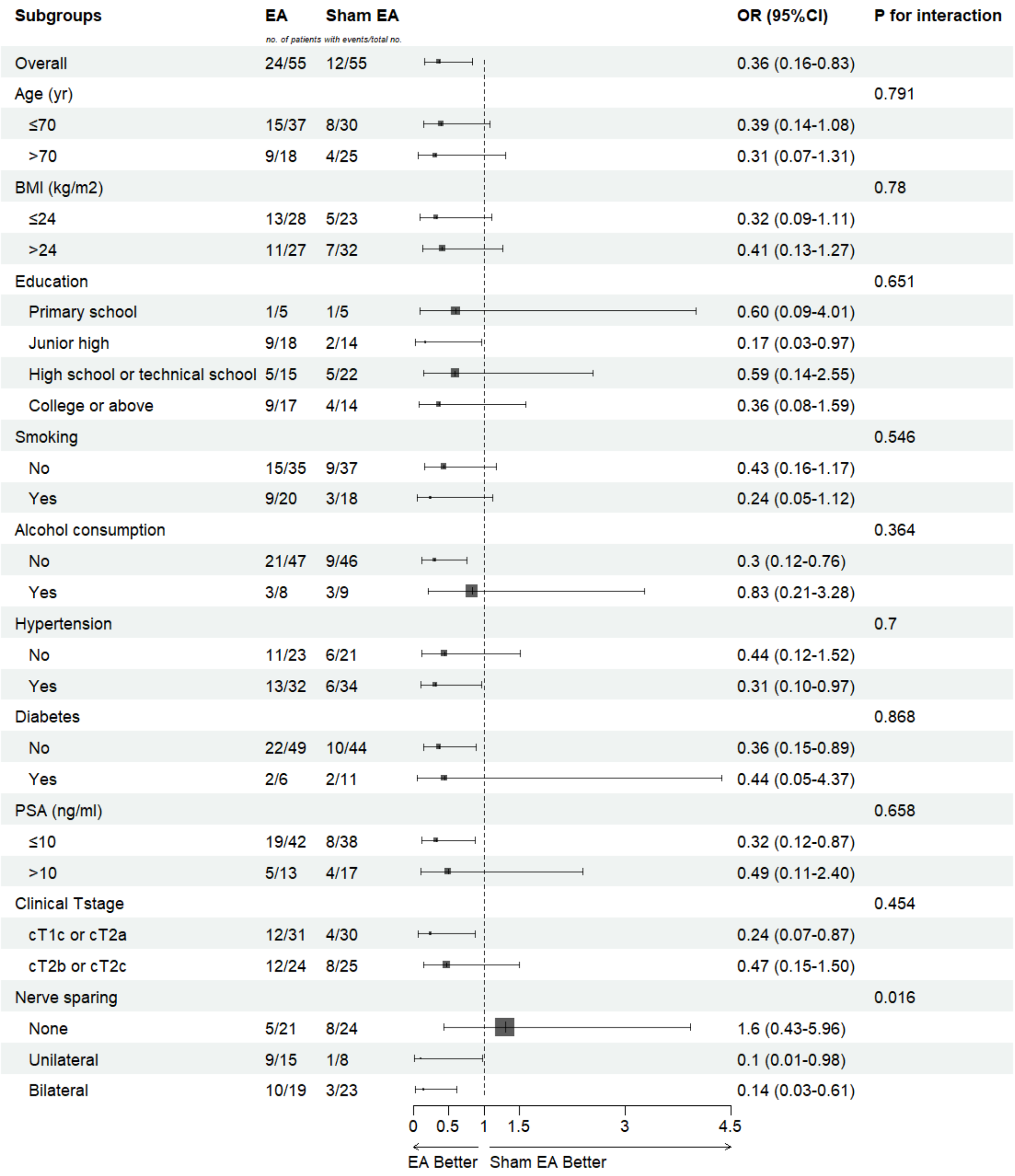

Abbreviations: EA, electroacupuncture; BMI, body mass index; PSA, prostate specific antigen; OR, odds ratio.

The sizes of the boxes represent the magnitude of OR.

© 2025 Niu J et al. *JAMA Network Open*.
