# Supplementary material for: Electroacupuncture in Patients With Early Urinary Incontinence After Radical Prostatectomy: A Randomized Clinical Trial
Source: JAMA Netw Open. 2025 Sep 30;8(9):e2534491. doi: 10.1001/jamanetworkopen.2025.34491 (PMC12485641; doi:10.1001/jamanetworkopen.2025.34491)
Supplement: Supplement 3. — Data Sharing Statement [file jamanetwopen-e2534491-s003.pdf]

Data Sharing Statement

Niu. Electroacupuncture in Patients With Early Urinary Incontinence After Radical Prostatectomy. *JAMA Netw Open*. Published September 30, 2025. doi:10.1001/jamanetworkopen.2025.34491

Data
Additional Information: name:The Study of Electrical Acupuncture Stimulation Therapy for Postprostatectomy Incontinence
URL:https://clinicaltrials.gov/study/NCT04972669?locn=Drum%20Tower%20Hospital,%20Medical%20School%20of%20Nanjing%20University&limit=25&term=Electroacupuncture&rank=2
registration number:NCT04972669
Data available: Yes
Data types: Deidentified participant data
How to access data: Proposals for data access should be directed to Xuefeng Qiu：Xuefeng_qiu@nju.edu.cn.
When available: beginning date: 01-30-2026, end date: 02-28-2026

Supporting Documents
Document types: Informed consent form
How to access documents: Informed consent can be accessed through connecting Xuefeng_qiu@nju.edu.cn.
When available: With publication

Additional Information
Who can access the data: Researchers whose proposed use of the data has been approved
Types of analyses: For a specific purpose
Mechanisms of data availability: After approval of a proposal, and with a signed data access agreement
